# Supplementary material for: Mapping epigenetic divergence in the massive radiation of Lake Malawi cichlid fishes
Source: Nat Commun. 2021 Oct 7;12:5870. doi: 10.1038/s41467-021-26166-2 (PMC8497601; doi:10.1038/s41467-021-26166-2)
Supplement: Supplementary file 3 — Description of Additional Supplementary File [file 41467_2021_26166_MOESM3_ESM.pdf]

Description of Additional Supplementary Files

File name: Supplementary Data 1
Description:
One excel file containing two spreadsheets summarising the sequencing sample IDs as well as the sequencing and mapping-related results for all WGBS (spreadsheet 1) and RNAseq (spreadsheet 2) samples. Mean and standard deviation (sd) values for each column are given at the bottom of each spreadsheet.

Spreadsheet 1 (WGBS) - column information:
ID: sample ID (short)
ID_Cam: internal sample ID
HiSeq lane: fraction of one HiSeq 2500/4000 flow cell lane used for each sample. <1 means multiplexing.
HiSeq: Illumina sequencer used
Species: full species name
Eco group: Ecomorphological group of each sample.
Sex: sample sex
Type: wild-caught or tank-reared sample
Info: additional information (related to fishing method and GPS coordinates of fishing location)
Tissue: tissue used to extract DNA (either liver or muscle)
HiSeq ID: full HiSeq ID for each sample (refer to GEO ID); some samples have been sequenced more than once (multiple sequencing files).
n_PEread_processed: number of paired-end sequencing reads generated for each sample.
p_bisulfite_conv.: bisulfite conversion rate (in percentage; calculated using unmethylated spiked-in lambda DNA; see Methods).
n_uniquePE_Mapped: unique best mapping rate of paired-end reads (% total reads; 0 mismatch allowed; aligned to SNP-corrected MZ genomes using Bismark and after TrimGalore filtering [see Methods]).
n_non-clonal_reads: read count of non-PCR duplicate reads.
NonClonalReads_left%: percentage of non PCR duplicate reads (usable reads).

Spreadsheet 2 (RNAseq) - column information:
ID_Cam: full sample ID (refers to GEO ID).
ID: internal short IDs.
Extraction: type of RNA extraction (total RNA for all)
Library: library type (ribosomal RNA depletion [RiboZero] for all)
Species names: full species name
tissue: tissues utilised to extract total RNA
Sex: sample sex
Diet: species diet
Habitat: species habitat
Location : Lake Malawi/Victoria cichlid species
Specimen_size: size of the species analysed.
n_processed: total count of paired-end sequencing reads generated.
n_pseudoaligned: number of reads pseudoaligned (using kallisto)
n_unique: number of uniquely aligned reads (using kallisto)
unmapped: number of unmapped reads (kallisto)
p_pseudoaligned: percentage of pseudoaligned reads (kallisto)
p_unique: percentage of uniquely aligned reads (kallisto)
