# Supplementary material for: Mapping epigenetic divergence in the massive radiation of Lake Malawi cichlid fishes
Source: Nat Commun. 2021 Oct 7;12:5870. doi: 10.1038/s41467-021-26166-2 (PMC8497601; doi:10.1038/s41467-021-26166-2)
Supplement: Supplementary file 5 — Reporting Summary [file 41467_2021_26166_MOESM5_ESM.pdf]

nature research

Corresponding author(s): E.A. Miska, G. Vernaz

Last updated by author(s): Aug 26, 2021

Reporting Summary

Nature Research wishes to improve the reproducibility of the work that we publish. This form provides structure for consistency and transparency in reporting. For further information on Nature Research policies, see our Editorial Policies and the Editorial Policy Checklist.

Statistics

For all statistical analyses, confirm that the following items are present in the figure legend, table legend, main text, or Methods section.

n/a	Confirmed	
☐	☒	The exact sample size (*n*) for each experimental group/condition, given as a discrete number and unit of measurement
☐	☒	A statement on whether measurements were taken from distinct samples or whether the same sample was measured repeatedly
☐	☒	The statistical test(s) used AND whether they are one- or two-sided<br>*Only common tests should be described solely by name; describe more complex techniques in the Methods section.*
☐	☒	A description of all covariates tested
☐	☒	A description of any assumptions or corrections, such as tests of normality and adjustment for multiple comparisons
☐	☒	A full description of the statistical parameters including central tendency (e.g. means) or other basic estimates (e.g. regression coefficient) AND variation (e.g. standard deviation) or associated estimates of uncertainty (e.g. confidence intervals)
☐	☒	For null hypothesis testing, the test statistic (e.g. *F*, *t*, *r*) with confidence intervals, effect sizes, degrees of freedom and *P* value noted<br>*Give P values as exact values whenever suitable.*
☒	☐	For Bayesian analysis, information on the choice of priors and Markov chain Monte Carlo settings
☒	☐	For hierarchical and complex designs, identification of the appropriate level for tests and full reporting of outcomes
☐	☒	Estimates of effect sizes (e.g. Cohen's *d*, Pearson's *r*), indicating how they were calculated

Our web collection on statistics for biologists contains articles on many of the points above.

Software and code

Policy information about availability of computer code

Data collection	No software was used to collect data.
Data analysis	TrimGalore v.0.6.2, bismark v.0.20.0, lastz v1.02, kallisto v0.46.0, samtools v1.9, bedtools v2.27.1, R v.3.6.2 (R packages: DSS v.2.32.0, pheatmap v1.0.12, ggplot2 v.3.3.0, tidyverse v1.3.0, FSA v0.8.25, sleuth v0.30.0,deepTools v3.2.1, phangorn v2.5.5), IGV v2.5.2, RepeatMasker v4.0.9.p2, RepeatModeler v1.0.11, makeCGI (v1.3.4), g:Profiler (version: e100_eg47_p14_7733820), UCSC liftOver tool (which includes scripts: axtChain and liftOver; kent source version 418), evo getWGSeq script from https://github.com/millanek/evo; v.0.1 r24, commit99d5b22 .

For manuscripts utilizing custom algorithms or software that are central to the research but not yet described in published literature, software must be made available to editors and reviewers. We strongly encourage code deposition in a community repository (e.g. GitHub). See the Nature Research guidelines for submitting code & software for further information.

Data

Policy information about availability of data

All manuscripts must include a data availability statement. This statement should provide the following information, where applicable:

- Accession codes, unique identifiers, or web links for publicly available datasets
- A list of figures that have associated raw data
- A description of any restrictions on data availability

All raw sequencing reads (SNP-corrected genomes, WGBS and RNAseq) have been deposited (GEO accession GSE158514; https://www.ncbi.nlm.nih.gov/geo/query/acc.cgi?acc=GSE158514). Sample ID numbers are listed in Supplementary Table 1. In addition, variant call files for all species of this study (for SNP-corrected genomes and pairwise whole genome sequence divergence), as well as RNAseq for A. calliptera tis-sues were downloaded from NCBI Short Read Archive BioProjects PRJEB1254 (https://www.ncbi.nlm.nih.gov/bioproject/?term=PRJEB1254) and PRJEB15289 (https://www.ncbi.nlm.nih.gov/bioproject/?term=PRJEB15289).

Field-specific reporting

Please select the one below that is the best fit for your research. If you are not sure, read the appropriate sections before making your selection.

☐ Life sciences ☐ Behavioural & social sciences ☒ Ecological, evolutionary & environmental sciences

For a reference copy of the document with all sections, see nature.com/documents/nr-reporting-summary-flat.pdf

Ecological, evolutionary & environmental sciences study design

All studies must disclose on these points even when the disclosure is negative.

Study description	This study aimed at generating high-coverage whole-genome bisulfite sequencing and total RNA sequencing of both liver and muscle tissues from male, wild-caught Lake Malawi cichlid species in order to study the epigenetic divergence in a massive vertebrate radiation.
Research sample	In total, we selected 6 different Lake Malawi cichlid species in order to assess transcriptome and methylome variation across 5 of the 7 eco-morphological groups observed in Lake Malawi cichlid radiation in our study. We also selected one Lake Victoria species as an outgroup (for transcriptome analysis).<br>The species selected were the following (in brackets are the distinct eco-morphological groups/populations each species belong to):<br>Maylandia zebra (Mbuna group, 2-3 biological replicates for liver and muscle tissues [both RNAseq and WGBS]),<br>Petrotilapia genalutea (Mbuna group, 2-3 biological replicates for liver and muscle tissues [both RNAseq and WGBS]),<br>Rhamphochromis longiceps (Rhamphochromis group, 2 biological replicates for liver and muscle tissues [both RNAseq and WGBS]),<br>Diplotaxodon limnothrissa (Diplotaxodon group, 2-3 biological replicates for liver and muscle tissues [both RNAseq and WGBS]),<br>Aulonocara stuartgranti sp. Usisya (Deep benthic group, 2-3 biological replicates for liver and muscle tissues [WGBS only]),<br>Astatotilapia calliptera sp. Itupi (Calliptera group, 2-3 biological replicates for liver and muscle tissues [both RNAseq and WGBS]),<br>Pundamilia nyrerei (Lake Victoria outgroup, 3 biological replicates for liver [RNAseq only]).<br>All fish were size-matched wild caught male specimens displaying full nuptial colorations (when males) and collected by collaborators and bought dead from fishermen. One female sample was analysed in the case of A.stuartgranti.
Sampling strategy	The main strategy was to select 2-3 biological replicates for each tissues of each Lake Malawi cichlid species spanning most of the eco-morphological groups featured in Lake Malawi cichlid radiation. No statistical method was used to define sample size - sample sizes were based on literature (2-3 biological replicates for WGBS and RNAseq analyses). Within-species variation for all analyses and statistical procedures was taken into account. All fish were size-matched wild caught male specimens displaying full nuptial colorations (when males) and collected by collaborators and bought dead from fishermen. One female sample was analysed in the case of A.stuartgranti.
Data collection	Fish were collected, identified (photographs) and registered in an excel sheet (see Supplementary Data 1) by GFT, MJG, MM, HS, MG, AMT. DNA extraction and WGBS library preparation were performed by GV. RNA extraction was performed by MD. Illumina HiSeq sequencing was performed by the sequencing facility at CRUK, Cambridge UK (WGBS) and by the sequencing facility of the Wellcome Sanger Institute (RNAseq). All sequencing data was analyzed by GV. SNP-corrected genomes were generated by MM.
Timing and spatial scale	Liver and muscle tissues for all Malawi Cichlid fish were dissected from wild dead fish by G. F. Turner, M. Malinsky, H. Svardal, A. M. Tyers, M. Mulumpwa and M. Du over the course of one field trip in 2016 (5 days in Feb 2016) in Malawi in collaboration with the Fisheries Research Unit of the Government of Malawi). The strategy was to collect all the samples at the same time. In the case of A.calliptera, tissues were collected in Feb 2015 (1 day; at the same time) in Tanzania by GFT and AMT in collaboration with the Tanzania Fisheries Research Institute (various collaborative projects). Upon collection, tissues were immediately placed in RNAlater (Sigma), and were then stored at -80°C upon return to the UK.
Data exclusions	No data were excluded.
Reproducibility	All attempts (4 attempts) to reproduce the results using raw sequencing data independently were successful.
Randomization	Species were selected based on their respective eco-morphological traits (using published literature). Randomization was not appropriate.
Blinding	All analyses pertaining to genome-wide methylome and transcriptome differences (including hierarchical clustering, principal component analysis) were performed in an unbiased, blind approach (Name species were not known) - this allowed us to identify potential outliers or false species/tissues identification (none were seen). For other analysis (DMR, DEG), investigators were aware of the species/groups identity (group/species/tissues allocation done based on species identification using photographs and unbiased clustering of RNAseq and WGBS data variation) in order to identify differentially expressed genes and differentially methylated regions between groups (species allocation had to be performed for those analyses) - biological replicates were used and within-species variation was taken into account for such analyses.

Did the study involve field work? ☐ Yes ☒ No

Reporting for specific materials, systems and methods

We require information from authors about some types of materials, experimental systems and methods used in many studies. Here, indicate whether each material, system or method listed is relevant to your study. If you are not sure if a list item applies to your research, read the appropriate section before selecting a response.

Materials & experimental systems

n/a	Involved in the study
☒	☐ Antibodies
☒	☐ Eukaryotic cell lines
☒	☐ Palaeontology and archaeology
☐	☒ Animals and other organisms
☒	☐ Human research participants
☒	☐ Clinical data
☒	☐ Dual use research of concern

Methods

n/a	Involved in the study
☒	☐ ChIP-seq
☒	☐ Flow cytometry
☒	☐ MRI-based neuroimaging

Animals and other organisms

Policy information about studies involving animals; ARRIVE guidelines recommended for reporting animal research

Laboratory animals	The study did not involve laboratory animals.
Wild animals	All Malawi cichlid fish were bought dead from local fishermen by G. F. Turner, M. Malinsky, H. Svardal, A. M. Tyers, M. Mulumpwa and M. Du in 2016 in Malawi in collaboration with the Fisheries Research Unit of the Government of Malawi), or in 2015 in Tanzania in collaboration with the Tanzania Fisheries Research Institute (various collaborative projects). Upon collection, tissues were immediately placed in RNAlater (Sigma), and were then stored at -80°C upon return. The species, sex and location of each sample are provided in SUPP Table 1.
Field-collected samples	No laboratory work with live animals was performed.
Ethics oversight	Sampling collection and shipping were approved by permits issued to GF Turner, MJ Genner R Durbin, EA Miska by the Fisheries Research Unit of the Government of Malawi (various collaborative projects) and the Tanzania Fisheries Research Institute.

Note that full information on the approval of the study protocol must also be provided in the manuscript.
